# Pulmonary arterial hypertension in Latin America: epidemiological data from local studies

**DOI:** 10.1186/s12890-018-0667-8

**Published:** 2018-06-26

**Authors:** Ana Beatriz Valverde, Juliana M. Soares, Karynna P. Viana, Bruna Gomes, Claudia Soares, Rogerio Souza

**Affiliations:** 1 Latin America Medical Department – GlaxoSmithKline, Estrada dos Bandeirantes, Rio de Janeiro, 8464 Brazil; 2 Pulmonary Hypertension Unit, Pulmonary Department – Heart Institute, University of Sao Paulo Medical School, Av. Dr. Eneas de Carvalho Aguiar, 44, Sao Paulo, 05403-000 Brazil

**Keywords:** Pulmonary arterial hypertension, Epidemiology, Prognosis, Latin America

## Abstract

**Background:**

Pulmonary arterial hypertension is a rare, progressive disease with poor prognosis. However, there is limited information available on the characteristics of PAH patients outside of North America and Europe. This is particularly important as researchers have described that there are potential geographical and regional differences which are vital to consider in the design of clinical trials as well as PAH treatment. The aim of this study was to describe the epidemiology of PAH (PH group 1) in Latin America.

**Methods:**

A search of electronic databases for studies published in English, Spanish or Portuguese was conducted specifying publication dates from the 1st of January 1987 until 10th October 2016. Two authors independently assessed papers for inclusion and extracted data. A narrative synthesis of the findings was conducted.

**Results:**

The search revealed 22 conference abstracts and articles, and on application of the inclusion criteria, six conference abstracts and articles were included in the final review. Studies/registries were based in Argentina, Brazil and Chile. In contrast to the available literature from developed countries, in Latin America, most patients were diagnosed at younger age; nevertheless, the higher prevalence of idiopathic PAH (IPAH) and the advanced stage of the disease at diagnosis were comparable to the existing literature, as the long term survival, despite the lower availability of targeted therapies.

**Conclusion:**

This study highlights the regional characteristics in the epidemiology of group 1 PH. The recognition of these differences should be considered when developing clinical guidelines and extrapolating diagnostic and treatment algorithms. Equitable access to health care and therapies are also issues that need to be addressed in Latin America. Information coming from a large prospective registry representing the different populations in Latin America is of critical importance to increase disease awareness in the region and improve diagnosis and management.

**Electronic supplementary material:**

The online version of this article (10.1186/s12890-018-0667-8) contains supplementary material, which is available to authorized users.

## Background

Pulmonary arterial hypertension (PAH), a clinical classification of group 1 pulmonary hypertension (PH), is a rare, progressive disease with poor prognosis. It has a worldwide estimated prevalence ranging from 10 to 16 cases per million inhabitants per year and an incidence between 2.0 to 3.2 cases per million inhabitants [1, 2]. In the last two decades, knowledge of the basic pathobiology of PAH, its natural history, prognostic indicators, and therapeutic options have improved. National registries have provided a better understanding of the epidemiology and clinical evolution of the disease [3] as well as valuable information on disease characteristics, demographics and outcomes of patients with PAH [4], allowing the development of risk stratification tools [5]. Two recent reviews [2, 6] have identified 11 PAH registries based in the United States (US), China, France, Scotland, United Kingdom (UK), Spain and a European Union consortium. However, limited information is available on the characteristics of PAH patients outside of North America and Europe. Moreover, no Latin American studies were included in these reviews. This is particularly important as researchers have described that there are potential geographical and regional differences which are vital to consider in the design of clinical trials as well as PAH treatment [2, 6, 7]. The aim of this study was to describe the epidemiology of PAH (group 1) in Latin America.

## Methods

To identify relevant studies, a search was conducted using Medline, PubMed, LILACS, EMBASE, SciElo, PAHO, BVS, Cochrane, Latindex, CAPES and Searchlight (a GlaxoSmithKline database that includes conference abstracts) specifying publication dates from the 1st of January 1987 until 10th October 2016. The search terms included “pulmonary arterial hypertension”, combined with “registry”, “cohort study” and “observational study”. A web-based search, using the Internet search engine ‘Google Scholar’, was also conducted.

Studies were included if they were based in Latin America (Central and South America) and the Caribbean [8], examined PAH (PH group 1) adult patients aged between 18 and 65 years old and were available in English, Spanish and/or Portuguese. Studies were required to report on at least one of the following topics: clinical characteristics (etiology, time from onset of symptoms to diagnosis, hemodynamic parameters and severity of the disease based on the World Health Organization – WHO – classification); demographic characteristics (age and gender); treatment pattern or survival rates in a cohort of PAH patients. Publications were excluded if they focused on a subgroup of PAH patients and not a cohort of all PAH or all IPAH patients, for example articles that investigated PAH only in pregnant women or pediatric patients, and/or PAH patients treated with a particular treatment. Additionally, studies that focused on patients with a specific etiology associated with group 1 PH such as schistosomiasis, HIV, lupus or coronary heart disease were also excluded. Due to the paucity of data, the decision was made to include conference abstracts if they have any publication describing the study design to assure the correct understanding of the data collection, patient inclusion, study results and methodology.

The following items were extracted from each article: inclusion and exclusion criteria; sample size; country where the study was conducted; study design; study population, period of enrollment and follow up; incidence and prevalence; diagnosis criteria; PAH patient’s demographic characteristics (age and gender); co-morbidities; time from onset of symptoms until the diagnosis; PAH etiologies; PAH survival and PAH treatment. Two reviewers independently extracted data using a standardized data extraction form.

## Results

A total of 22 publications including articles and conference abstracts were retrieved by the literature search. Fourteen conference abstracts were screened. From these, twelve were excluded. The Mexican registry [9], two abstracts that reported data from the Colombian registry [10, 11] and a study from Puerto Rico [12] were excluded as they did not report data separately for group 1 PH patients. The Paraguayan registry [13] was excluded as data was reported according to the treatment received by each patient’s group (for example, group A: patients only treated with Sildenafil). Five conference abstracts were excluded as they reported on the same results from the **HI**pertensió**N PUL**monar y A**S**ociaciones en la **AR**gentina (HINPULSAR) registry [14–18]. Also, one publication from the **Re**gistro **Co**laborativo de Hi**p**ertens**i**ón Pu**l**monar en Argentina (RECOPILAR) registry [19] was excluded because it reported duplicate data. Additionally, one registry from Uruguay [20] was excluded as they did not provide another publication detailing the study design.

Two conference abstracts, one from the HINPULSAR registry [21] and one from RECOPILAR registry [22], both based in Argentina were included in the final analysis. The data from the abstracts was complemented with methodological information from the study protocols [23, 24].

**Fig. 1:**
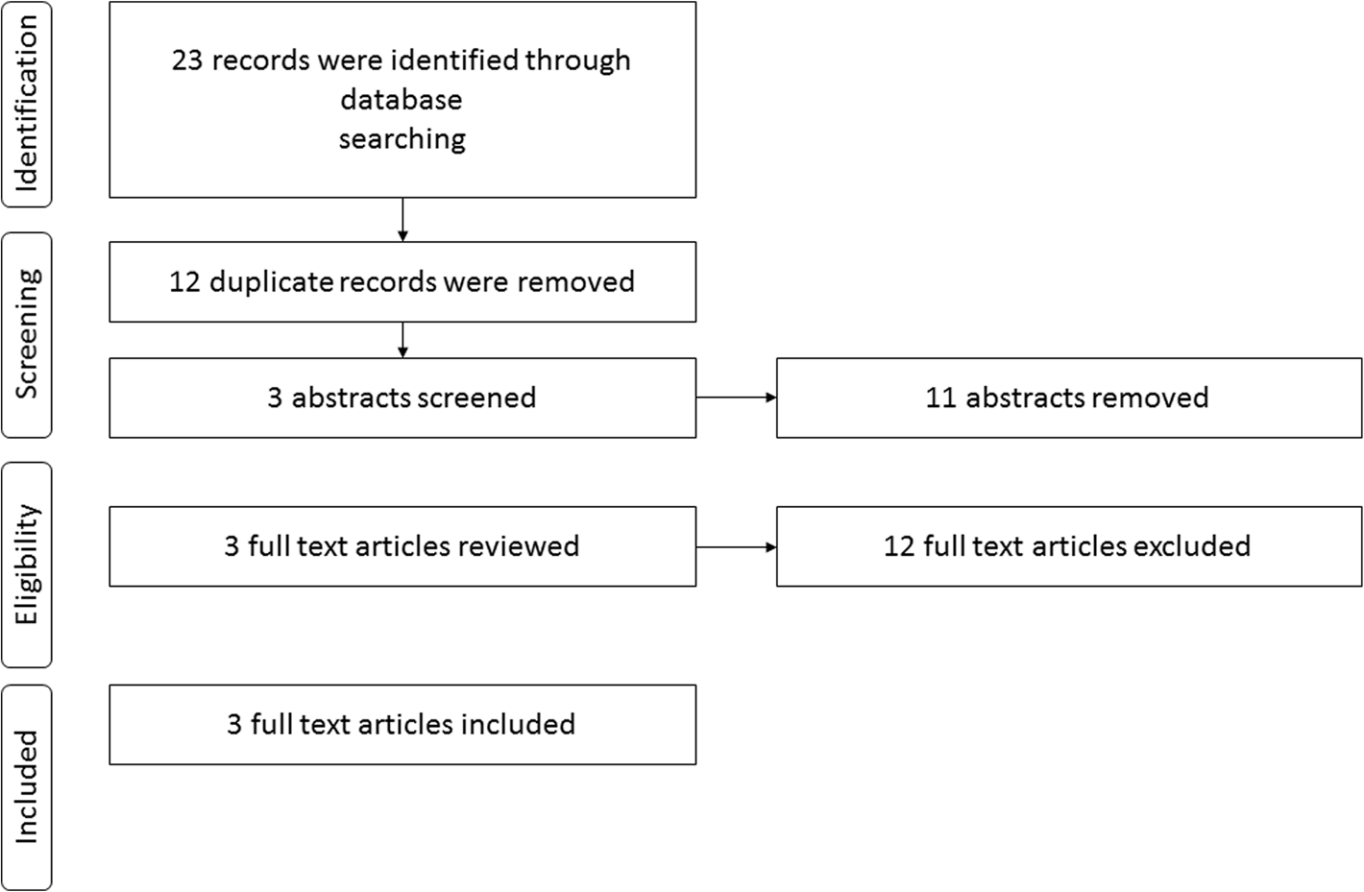
Inclusion and exclusion criteria

Eight full text articles were found of which four were excluded from the analysis. One study conducted in Brazil [25] was excluded because unlike the other studies, PH diagnosis was made based only on echocardiography results and did not consider hemodynamic parameters. The other study from Chile [26] was excluded as the study included group 1 and 4 PH patients but did not report data separately for group 1 PH. The other two excluded publications were conducted in Argentina and described only the study protocol and methodology of the HINPULSAR [23] and the RECOPILAR registries [24] but did not report results. Four articles were included in the final analysis: two Chilean [27, 28] one Argentinean [29] and one Brazilian [7]. The characteristics of the PAH registries are provided in Table 1. In total, six publications (two conference abstracts and four full articles) were qualified for inclusion according to the eligibility criteria (see Fig. 1). The publications excluded are described in Additional file 1.

**Table 1:** Characteristics of PAH registries/studies included in the review

Characteristic	Argentina [29]	Brazil [7]	Chile [28]	HINPULSAR [21]	RECOPILAR [22]	Chile [27]
Study design and time period	Prospective January 2004–March 2012	Prospective January 2008–December 2013	1999–2005	Prospective January 2010–December 2011	Prospective July 2014 – May 2015	Prospective June 2003–March 2005
Number of centres	1	1	2	31	Multicenter^a^	1
Study cohort	Group 1 PH	Group 1 PH	Group 1 PH	Group 1 PH	Group 1 PH	Group 1 PH and Group 4 PH
Percentage of patients with group 1 PH (number of PAH patients)	100% (125)	100% (178)	100% (17)	100% (124)	100% (170)	93% (27)
% IPAH patients	49	29	80	52	52	41
% CTD-PAH	14	26	13	15	15	26
% CHD-PAH	28	8	_	27	27	33
% Sch-PAH	_	20	_	_	_	_
% Others^b^	9	18	7	6	6	_
% female	79	77	60	78	79	86
Mean age (years-old)	34 ± 16	46 ± 15	45	45 ± 17	51	41 ± 14
% FC III/IV	58	46	47	62	70	85
6MWD (m)	360	383 ± 152	348 ± 98	_	373	378 ± 113
RAP (mm Hg)	8	10 ± 5	12 ± 8	_	10	8 ± 7
mPAP (mm Hg)	54	52 ± 18	57 ± 15	55 ± 20	_	59 ± 12
PVR (woods units)	12	10 ± 6	_	_	_	_
CI (L/min/m^2^)	2	3 ± 1	2 ± 1	_	3	3 ± 1
Time from onset of symptoms until diagnosis (years)	1.4	_	_	_	_	2.9

*CTD* connective tissue disease, *CHD* congenital heart disease, *Sch* schistosomiasis-associated, *FC* functional class, *6MWD* 6-minute walking distance, *RAP* right atrial pressure, *mPAP* mean pulmonary artery pressure, *PVR* pulmonary vascular resistance, *Cl* cardiac index

^a^The number of centres was not provided

^b^Others: PAH associated to drugs and toxins, associated to HIV and portal hypertension

All studies were conducted in South America, mainly in Argentina, Brazil and Chile. The number of patients with group 1 PH varied from 17 in Chile [28] to 178 in Brazil [7]. The number of centres involved in the registries/studies varied from 1 to 31 [21]. The studies found did not report on or calculate the incidence and prevalence of PAH in Latin America (see Table 1).

The mean age of PAH patients in Latin America varied from 34 [29] to 51 years [22]. All the studies included just adult patients, except the Argentinean by Talavera et al., [29] that included 16 patients (12.8%) younger than 18 years. All studies reported greater frequency of PAH in female patients ranging from 60% [28] up to 86% [27], both values found in small cohorts in Chile.

**Fig. 2:**
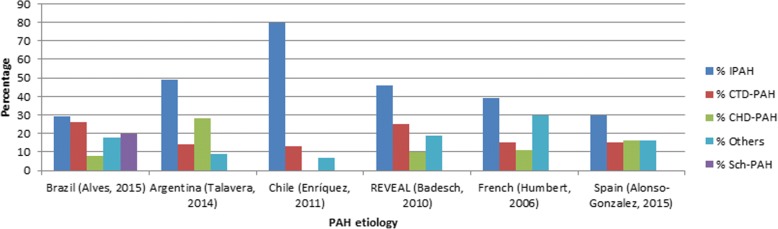
PAH etiologies: differences between Latin America and other international registries

The most commonly reported subtype among PAH patients was IPAH. One of the Chilean studies [28] showed the highest percentage of IPAH patients (80%) and the Brazilian registry [7] had the lowest with 29%. However, the Brazilian registry [7] mentioned schistosomiasis as one of the most common subtypes of PAH (Sch-PAH in 20% of total patients). As can be seen in Fig. 2, the percentage of IPAH patients is higher in Latin America compared to European studies and the REVEAL registry from the United States (US).

All the studies defined PAH as the presence of mean pulmonary arterial pressure (mPAP) greater than 25 mmHg at rest and a pulmonary artery wedge pressure (PCWP) less than 15 mmHg after right heart catheterization (RHC) [30]. Only one of the Argentinean studies [29] and one of the Chilean studies [27] reported the time from onset of symptoms to diagnosis (1.4 and 2.9 years, respectively).

The Brazilian registry [7] reported the lowest proportion of patients in the New York Heart Association (NYHA)/WHO functional class III or IV (46%). The Argentinean registries reported similar proportion of patients with functional class III or IV (ranging from 58 to 70%) [21, 22, 29]. One Chilean study [27] with 27 group 1 PH patients showed the highest proportion of patients with severe functional class (85%). Despite the differences, these values are considered high and demonstrate that most of the patients were in an advanced stage of the disease. Regarding the 6-min walk distance (6MWD), in general, the studies exhibited the same pattern for exercise capacity, ranging from 348 m in Chile [28] up to 383 m in Brazil [7].

The hemodynamic parameters exhibited the same pattern in all studies. Mean right atrial pressure (RAP) ranged from 8 in the Chilean [27] and Argentinean [29] registries up to 12 ± 8 mmHg in the Chilean study [28]. The mean pulmonary artery pressure (mPAP) ranged from 52 ± 18 mmHg in the Brazilian study [7] up to 59 ± 12 mmHg in Chile [27]. Regarding the pulmonary vascular resistance (PVR), only the Brazilian [7] and the Argentinean [29] registries reported this parameter and it was similar (10 ± 6 and 12 woods units, respectively). The studies also exhibited the same pattern for cardiac index (CI) (See Table 1).

**Fig. 3:**
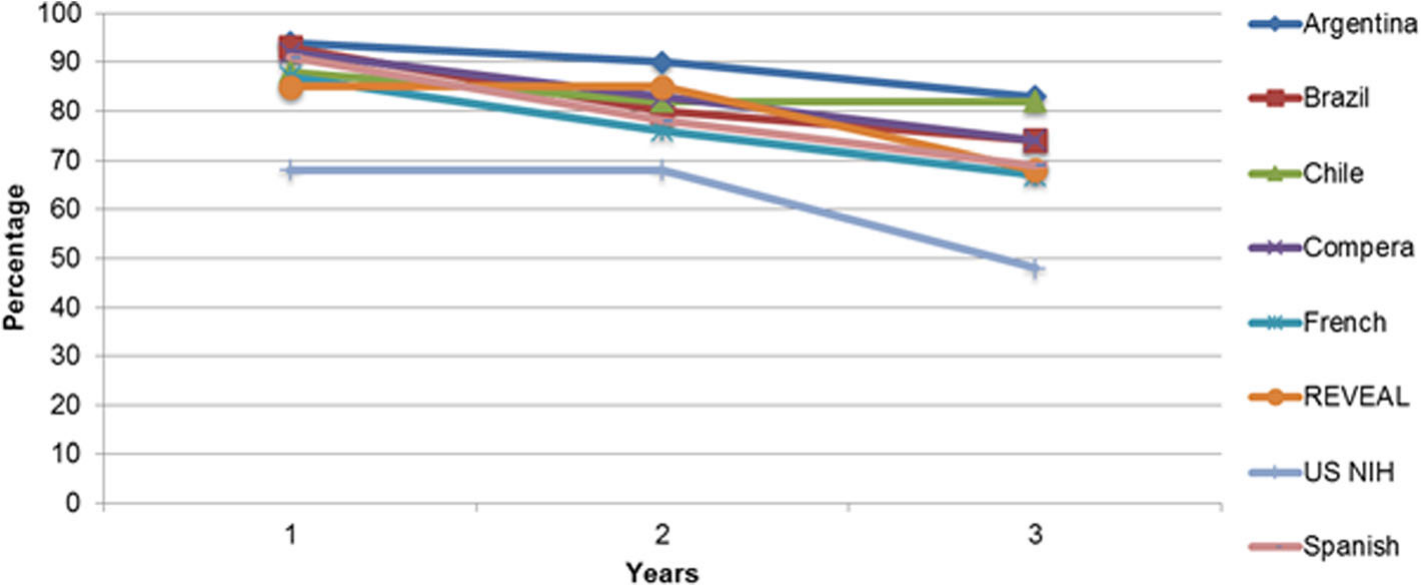
Survival rates (%) in 1, 2 and 3 years in Latin America registries compared to international registries

Three studies reported the survival rates at 1, 2 and 3 years after diagnosis. The Brazilian registry [7] described survival only for incident patients and the Argentine study [29] showed survival for incident and prevalent patients. The Chilean study [28] did not specify if they included incident and/or prevalent patients. Comparing the Brazilian [7] and Argentinean [29] registries in the first year, the survival was similar (92.7 and 94% respectively), but in the second (79.6% vs 90%) and third year (73.9% vs 83%), the survival of the Argentinean cohort was higher. The Chilean study by Enríquez et al. [28] with a small sample size (*N* = 17) showed at first year a 88% of survival and the same survival rate at years 2 and 3 (82%). Figure 3 shows the survival rates in these Latin American studies compared to other international registries.

Most of the studies described the treatment received by patients [7, 16, 28, 29]. The Argentinean [29] and the Brazilian [7] studies reported that nearly 30% of patients were treated with Bosentan. It is important to note that the Brazilian registry only included incident patients [7] and hence only first-line treatment. On the other hand, the HINPULSAR and the Chilean registries reported that only 12% of the patients were treated with Bosentan. Sildenafil was considered as first line treatment in Argentina and Brazil [7]. The highest percentage of Sildenafil use was in Argentina [29] (83%) and lowest in Chile [28] (24%). However, the Chilean registry [28] reported the highest percentage of patients receiving treatment with Ambrisentan (82%). Inhaled Iloprost use was mentioned in HINPULSAR [21] (11%), in the Argentinean registry [29] (32%) and also in Chile [28] (29%). This is despite the fact that in Chile the use of inhaled Iloprost was approved in 2005, when the study was finished and Ambrisentan was first approved in 2014, several years after the recruitment period of this study.

## Discussion

To our knowledge, this is the first article to describe the epidemiology of PAH (group 1 PH) in Latin America. As previously described, there is limited data from Latin America making it difficult to understand the disease and patient’s characteristics in the region as a whole. While registries are an instrumental source of information regarding the epidemiology and outcomes, they can be influenced by external factors related to local circumstances such as access to health care, disease awareness and living conditions [5, 31].

While there was variation in the average age among the Latin American countries, most patients diagnosed were young and of working age. As previously mentioned, the Argentinean study by Talavera et al. [29] had a lower mean age due to the fact that 16 patients aged under 18 years old were included in the registry. This is in contrast with results from developed countries where patients were older at diagnosis. The mean age of patients in the Giessen Pulmonary Hypertension Registry [30], the French registry [32], the US Registry to Evaluate Early and Long-Term PAH disease management (REVEAL) [33] and the Comparative, Prospective Registry of Newly Initiated Therapies for Pulmonary Hypertension (COMPERA) [34] was higher (≥ 50 years). Hoeper et al. [5], noticed that differences between countries may be explained by population age distribution (older population in Europe and US) and health care systems. However, other factors may play a role such as: referral patterns, PAH awareness, increase patient access to information and widespread use of noninvasive screening tools [6]. As noted by McGoon et al. [6], phenotypes may be related to the healthcare environment rather than to different expressions of the disease. Similar to the results of international registries, the prevalence of PAH in female patients was higher [2, 6].

The studies reviewed described differences in the prevalence of IPAH. For example, in Brazil the percentage of IPAH was lower but this could be explained by a high percentage of other etiologies such as the Sch-PAH. According to WHO, schistosomiasis affects more than 200 million people worldwide [7]. Estimates indicate that 8 to 12 million people are infected by schistosomiasis in Brazil [31], suggesting that schistosomiasis could be one of the main causes of PAH in the country. It is noteworthy that the proportion of PAH patients with congenital heart disease (CHD-PAH) reported in Argentina [29] (28%) was higher than what was described in Europe [32, 35] and North America [33] (below 15%). A recent review [35] emphasized the remarkable differences that might exist in specific areas of the world, as schistosomiasis in Brazil, or HIV in Africa, that should not be neglected when developing health policies for the appropriate diagnosis and management of PAH.

Functional class is a powerful predictor of outcomes in patients with PAH [36]. The majority of patients in the studies were in NYHA/WHO functional class III or IV. Patients in the Brazilian registry of incident cases [7] had lower proportion NYHA/WHO III/IV (46%), compared to most international (> 50%) [2, 6] and other Latin America studies [21, 22, 27]. It appears NYHA/WHO III/IV is higher in the US and Europe [2, 6] compared to Latin America (See Table 1). This is still the case when studies consider only incident or both prevalent and incident cases. However, even lower than in US and Europe, the percentage of patients in advanced functional class is still very high in Latin America, evidencing that patients are still diagnosed at late stages suggesting a lack of disease awareness and limited access to health care.

Hemodynamic parameters such as RAP, mPAP and PVR were similar to those reported in other international registries [2, 6]. The mean 6MWD in the Latin American studies (See Table 1) was higher compared to US and European registries [2, 6]. However, it is important to consider that the mean age of patients was lower in Latin America, which could contribute to a better 6MWD. Compared to older patients, younger patients (< 50 years) have a shorter duration of symptoms, fewer comorbidities associated, better exercise capacity, and despite more severe hemodynamic impairment, better survival [2]. As previously noted, the percentage of patients in advanced functional class III/IV in Latin America was lower than in other regions, which may also contribute to a better exercise capacity. Alves et al. [7], have hypothesized that intrinsic characteristic of the patients or perhaps environmental factors associated with the socioeconomic conditions may also influence the level of daily activity of these patients. For example, patients may need to walk and/or travel more to reach the treating hospital.

Three studies reported survival rates. The Argentine registry had the highest 3-year survival rate [29]. The Brazilian study with only incident patients showed a high survival rate in the first year but in the second and third years the survival rate decreased [7]. As noted by McGoon et al. [6] and demonstrated by different studies, as the French and the Giessen registries [30, 31], compared to incident cases, prevalent ones had a better prognosis which could explain the differences between these studies. In general, Latin American patients had similar survival rates as patients in developed countries. The French [32] and REVEAL [33] studies exhibited a survival rate lower than the Argentinean [29] and Brazilian studies [7], despite the lower availability of targeted therapies in Latin America. A recent analysis of the COMPERA registry divided the PAH group into typical and atypical PAH, according to the presence of 3 or more risk factor for the existence of left heart disease, characterizing the atypical subgroup [37]. Patients with typical PAH were younger, without any remarkable difference in the hemodynamics profile. Although with similar overall survival, the response to treatment was higher in the subgroup with typical PAH. The study suggests that the presence of comorbidities might significantly influence the spectrum of PAH disease by adding different pathophysiological mechanisms to the more isolated vascular disease seen in the typical PAH. The lower mean age evidenced in Latin American patients suggests a lower prevalence of comorbidities which could contribute to a better survival rate in the region. Nevertheless, the lack of appropriate description of the comorbidities in the selected studies prevented a proper evaluation of the role of typical and atypical PAH prevalences in the overall survival.

Despite the fact that data on PAH treatment in Latin America is limited, oral drugs appear to be the main form of first line therapy with Sildenafil being the most commonly used drug for PAH treatment within the region. The lack of data on combination therapy may be due to the fact that it is not approved in most Latin American countries. It is also important to point out that some treatments that were reported in the studies have not been approved for PAH use in the countries where data was collected. This highlights the fact that entering a clinical trial may be one way of providing PAH patients an opportunity to receive specific treatment [31]. Timely and improved access to medicines may still be limited in the region. Efforts should be made to improve early diagnosis and the availability of new treatments which in turn may increase survival rates of PAH patients in Latin America.

Our study has limitations that need to be acknowledged. There is a clear paucity of available data regarding PAH in the region. Most of the PAH data in Latin America is available only in conference abstracts, making it difficult to evaluate the profile of PAH patients among the region. Research from non-English speaking countries is underrepresented in high-impact medical journals and indexation problems for journals in Spanish and Portuguese hinder the screening of studies.

While national registries are currently being implemented in different Latin American countries, accurate epidemiologic information on PAH is still limited. However, a new international multicenter registry “Registro Latinoamericano de Hipertensión Pulmonar (RELAHP)” was launched in 2014. This registry has been designed to collect medical history, diagnostic methods and treatment of patients suffering from pulmonary hypertension (PH) under optimal medical care in an effort to better fill the existing gap on the knowledge about the broader distribution of PAH in the region. Although there are some local registries in progress, more efforts and investments are still needed to ensure the dissemination of PAH data in Latin America.

## Conclusion

This study highlights the regional differences in the epidemiology of PAH. In contrast to Europe and North America, there is a clear heterogeneity in the distribution of the PAH forms in Latin America and the profile of patients described in the regional registries seems to be different from international ones. The recognition of these differences should be considered when developing clinical guidelines and extrapolating diagnostic and treatment algorithms. Specific health policies should address these differences while taking into account the limited health care access in some regions within Latin America. Equitable access to health care and therapies are issues that need to be addressed in Latin America. Information coming from a large prospective registry representing the different populations in Latin America is of critical importance to increase disease awareness in the region and improve diagnosis and management.

## Additional file

Additional file 1: **Table S1.** Characteristics of PAH registries/studies excluded from the review. (DOCX 71770 kb)

## Abbreviations

6MWD: 6-minute walking distance
CHD: Congenital heart disease
Cl: Cardiac index
COMPERA: Comparative, prospective registry of newly initiated therapies for pulmonary hypertension
CTD: Connective tissue disease
FC: Functional class
HINPULSAR: HIpertensión Pulmonar y Asociaciones en la Argentina
IPAH: Idiopathic pulmonary arterial hypertension
mPAP: Mean pulmonary artery pressure
NYHA: New York heart association
PAH: Pulmonary arterial hypertension
PCWP: Pulmonary artery wedge pressure
PH: Pulmonary hypertension
PVR: Pulmonary vascular resistance
RAP: Right atrial pressure
RECOPILAR: Registro Colaborativo de Hipertensión Pulmonar en Argentina
RELAHP: Registro Latinoamericano de Hipertensión Pulmonar
REVEAL: Registry to evaluate early and long-term PAH disease management
Sch: Schistosomiasis
UK: United Kingdom
US: United States of America
WHO: World Health Organization

## References

[1] Frost AE, Badesch DB, Barst RJ, Benza RL, Elliott CG, Farber HW (2011). The changing picture of patients with pulmonary arterial hypertension in the United States: how REVEAL differs from historic and non-US contemporary registries. Chest.

[2] Jiang X, Jing ZC (2013). Epidemiology of pulmonary arterial hypertension. Curr Hypertens Rep.

[3] Humbert M, Khaltaev N, Bousquet J, Souza R (2007). Pulmonary hypertension - from an orphan disease to a public health problem. Chest.

[4] McLaughlin VV, Shah SJ, Souza R, Humbert M (2015). Management of Pulmonary Arterial Hypertension. J Am Coll Cardiol.

[5] Hoeper MM, Simon RGJ (2014). The changing landscape of pulmonary arterial hypertension and implications for patient care. Eur Respir Rev.

[6] McGoon MD, Benza RL, Escribano-Subias P, Jiang X, Miller DP, Peacock AJ (2013). Pulmonary arterial hypertension: Epidemiology and Registries. J Am Coll Cardiol.

[7] Alves JL Jr, Gavilanes F, Jardim C, Fernandes CJCDS, Morinaga LTK, Dias B, et al. Pulmonary arterial hypertension in the southern hemisphere: results from a registry of incident brazilian cases. Chest. 2015;147(2):495–501. https://doi.org/10.1378/chest.14-1036. 10.1378/chest.14-1036 25317567

[8] United Nations. Standard country or area codes for statistical use (M49) - Methodology Geographic Regions Latin America and the Caribbean New York: The United Nations Statistics Division; 2017. Available from: https://unstats.un.org/unsd/methodology/m49/. Cited 16 2017 June

[9] Ramirez-Rivera A, Sanchez CJ, Garcia Badillo EV, Medellin B, Rivera SR, Palacios JM (2010). Northeast mexican registry on pulmonary arterial hypertension (RENEHAP). Chest.

[10] Conde R, Villaquiran C, Duenas R, Torres A. Diagnosis and Treatment of Pulmonary Arterial Hypertension and Chronic Thromboembolic Pulmonary Hypertension in Five Reference Centers In Bogota-Colombia, at 2.640 Meters Above Sea Level. Am J Respir Crit Care Med. 2015;191:A4847.

[11] Villaquiran C, Duenas R, Conde R, Torres A. Description of the Clinical, Functional and Hemodynamic Characteristics of Patients with Pulmonary Arterial Hypertension in Five Reference Centers in Bogota - Colombia, at 2.640 Meters Above Sea Level. Am J Respir Crit Care Med. 2015. 191; 2015:A3842

[12] Aranda A, Martin E, Fernandez R, Nieves J, Basora J, Torrellas P (2014). Puerto Rico pulmonary artery hypertension registry scheme. Chest.

[13] Chamorro F, Medina D, Melgarejo G (2015). Clinical management of pulmonary arterial hypertension. Eur J Heart Fail.

[14] Perna ER, Coronel ML, Echazarreta D, Cursack G, Marquez LL, Alvarez S (2012). The epidemiology of pulmonary arterial hypertension in HINPULSAR Registry showed areas for intervention in Argentina: promote early identification, improve the diagnostic strategy and treatment. Eur Heart J.

[15] Coronel M, Perna E, Echazarreta D, Lema L, Zini GP, Aristimuno G (2012). Treatment of pulmonary arterial hypertension according with functional class in the Argentinean HINPULSAR registry. Eur J Heart Fail.

[16] Coronel M, Perna E, Echazarreta D, Lema L, Zini GP, Aristimuno G (2012). Pulmonary arterial hypertension in Argentina: insights from HINPULSAR registry. Circulation.

[17] Echazarreta D, Coronel ML, Perna ER, Colque R, Cursack G, Nunez C (2012). Characterization of pulmonary hypertension and associations in Argentina: results of HINPULSAR registry. Eur J Heart Fail.

[18] Coronel ML, Perna ER, Nunez C, Cursack G, Fleitas M, Botta C (2014). Severe right ventricular dysfunction in pulmonary arterial hypertension: prevalence, clinical markers and treatment in Argentinean HINPULSAR registry. Eur J Heart Fail.

[19] Perna ER, Coronel ML, Diez M, Atamanuk N, Nitsche A, Caneva J (2016). First collaborative registry of pulmonary hypertension in Argentina (RECOPILAR registry): a clinical snapshot from a developing country. Eur J Heart Fail.

[20] Gruss AI, Pascal G, Salisbury JP, Trujillo P, Grignola JC, Curbelo P. Uruguayan National reference center in pulmonary hypertension: a population descriptive study. American Thoracic Society 2014 International Conference; San Diego: Am J Respir Crit Care Med. 2016. p. A4705.

[21] Perna ER, Coronel ML, Echazarreta D, Cursack G, Marquez LL, Alvarez S, et al. Epidemiological profile of pulmonary arterial hypertension in Argentina: insights from HINPULSAR registry. Eur J Heart Fail. 2012;SUPPL 1: S55.

[22] Lescano A, Talavera L, Mazzei J, Barimboim E, Saurit V, Varela B (2016). The advanced functional class and the variables of poor prognosis in pulmonary hypertension. Eur J Heart Fail.

[23] Federacion Argentina de Cardiologia - Comité de Insuficiencia Cardíaca e Hipertensión Pulmonar (2010). Diseño del Registro HINPULSAR: HIpertensióN PULmonar y aSociaciones en la ARgentina. Insuficiencia cardíaca.

[24] Echazarreta D, Perna E, Coronel MI (2014). Registro Colaborativo de Hipertensión Pulmonar en Argentina (RECOPILAR). Rev Fed Arg Cardiol.

[25] Lapa MS, Ferreira EVM, Jardim C, Martins BCS, Arakaki JSO, Souza R (2006). Características clínicas dos pacientes com hipertensão pulmonar em dois centros de referência em São Paulo. Revista da Associação Médica Brasileira.

[26] Herrera S, Gabrielli L, Paredes A, Saavedra R, Ocaranza MP, Sepúlveda P (2016). Sobrevida a mediano plazo en los pacientes con hipertensión arterial pulmonar en la era de terapias vasodilatadoras específicas del territorio vascular pulmonar. Rev Med Chil.

[27] Zagolin BM, Wainstein GE, Uriarte G de CP, Parra RC (2006). [Clinical, functional and hemodynamic features of patients with pulmonary arterial hypertension]. Caracterizacion clinica, funcional y hemodinamica de la poblacion con hipertension pulmonar arterial evaluada en el Instituto Nacional del Torax. Rev Med Chil.

[28] Enriquez A, Castro P, Sepulveda P, Verdejo H, Greig D, Gabrielli L (2011). Changes long term prognosis of 17 patients with pulmonary artery hypertension. Rev Med Chil.

[29] Talavera ML, Cáneva JO, Favaloro LE, Klein F, Boughen RP, Bozovich GE (2014). Hipertensión arterial pulmonar: Registro de un centro de referencia en Argentina. Rev Am Med Respir.

[30] Costa E, Jardim C, Bogossian H, Amato M, Roberto C, Carvalho R (2005). Acute vasodilator test in pulmonary arterial hypertension: evaluation of two response criteria. Vasc Pharmacol.

[31] Lopes AA, Bandeira AP, Flores PC, Santana MV (2010). Pulmonary hypertension in Latin America: pulmonary vascular disease: the global perspective. Chest.

[32] Humbert M, Sitbon O, Chaouat A, Bertocchi M, Habib G, Gressin V (2006). Pulmonary arterial hypertension in France: results from a national registry. Am J Respir Crit Care Med.

[33] Badesch DB, Raskob GE, Elliott CG, Krichman AM, Farber HW, Frost AE (2010). Pulmonary arterial hypertension: baseline characteristics from the REVEAL registry. Chest.

[34] Hoeper MM, Huscher D, Ghofrani HA, Delcroix M, Distler O, Schweiger C (2013). Elderly patients diagnosed with idiopathic pulmonary arterial hypertension: results from the COMPERA registry. Int J Cardiol.

[35] Alonso-Gonzalez R, Lopez-Guarch CJ, Subirana-Domenech MT, Ruíz JMO, González IO, Cubero JS (2015). Pulmonary hypertension and congenital heart disease: an insight from the REHAP National Registry. Int J Cardiol.

[36] Galiè N, Humbert M, Vachiery J-L, Gibbs S, Lang I, Torbicki A (2016). 2015 ESC/ERS Guidelines for the diagnosis and treatment of pulmonary hypertensionThe Joint Task Force for the Diagnosis and Treatment of Pulmonary Hypertension of the European Society of Cardiology (ESC) and the European Respiratory Society (ERS): Endorsed by: Association for European Paediatric and Congenital Cardiology (AEPC), International Society for Heart and Lung Transplantation (ISHLT). Eur Heart J.

