# Supplementary material for: Pulmonary arterial hypertension in Latin America: epidemiological data from local studies
Source: BMC Pulm Med. 2018 Jun 26;18:106. doi: 10.1186/s12890-018-0667-8 (PMC6019295; doi:10.1186/s12890-018-0667-8)
Supplement: Supplementary file 1 — Table S1. Characteristics of PAH registries/studies excluded from the review. (DOCX 71770 kb) [file 12890_2018_667_MOESM1_ESM.docx]

**Additional file 1: Table S1. Characteristics of PAH registries/studies excluded from the review**

| Characteristic | Brazil [25] | Chile [26] | Paraguay [13] | Mexico [9] | Colombia | Puerto Rico [12] | Uruguay [20] |
| --- | --- | --- | --- | --- | --- | --- | --- |
| Study design & time period | Retrospective January 1999 - August 2003 | Retrospective. 1999 – 2014 | Prospective. January 2012 – December 2014 | Prospective. 2004 - 2010 | Observational Retrospective. January 2008 – June 2014 | Prospective  July 2012 - July 2013 | Retrospective. June 2005 – June 2013 |
| Number of centres | 2 | 2 | 1 | 2 | 5 | N/A^a^ | 1 |
| Study cohort | Group 1 PH | Group 1 PH and Group 4 PH | Group 1 PH | Group 1 PH and Group 4 PH | Group 1 PH and Group 4 PH | Group 1 PH and Group 3 PH | Group 1 PH and Group 4 PH |
| Percentage of patients with group 1 PH (number of patients) | 100% (123) | 80% (96) | 100% (44) | 90% (108) | 67% (107) | 70% (40) | 52% (36) |
| % IPAH patients | 50 | 49 | Data divided by treatment groups. | 34 | 38 | _ | _ |
| % CTD-PAH | 11 | 20 |  | 22 | 32 | _ | 28 |
| % CHD-PAH | _ | 23 |  | 31 | 24 | _ | 43 |
| % Sch-PAH | 30 | _ |  | _ | _ | _ | _ |
| % Others^b^ | 9 | 3 |  | 4 | 6 | _ | _ |
| % female | 70 | 85 |  | 76 | 72 | 65 | 81 |
| Mean age (years-old) | 42 ± 14 | 44 ± 16 |  | 36 ± 14 | 50 ± 16 | _ | 47 ± 15 |
| % FC III/IV | 62 | 44 |  | _ | _ | 38 | 52 |
| 6MWD (m) | _ | 369 ± 119 |  | 414 ± 101 | 395 ± 152 | 274 | 426 ± 107 |
| RAP (mm Hg) | _ | 12 ± 7 |  | _ | _ | _ | _ |
| mPAP (mm Hg) | _ | 55 ± 15 |  | 61 ± 21 | 62 ± 23 | _ | 58 ± 10 |
| PVR (woods units) | _ | 13 ± 7 |  | _ | 12 ± 8 | _ | 12 ± 7 |
| CI (L/min/m^2^) | _ | 2 ± 1 |  | _ | 3 ± 1 | _ | 3 ± 1 |
| Time from onset of symptoms until diagnosis (years) | 1.7 | 1.4 | _ | _ | 2.6 | _ | 2.6 |

^a^The number of centres was not provided. ^b^Others: PAH associated to drugs and toxins, associated to HIV and portal hypertension. CTD: connective tissue disease, CHD: congenital heart disease, Sch: schistosomiasis-associated, FC: functional class, 6MWD: 6-minute walking distance, RAP: right atrial pressure, mPAP: mean pulmonary artery pressure, PVR: pulmonary vascular resistance, Cl: cardiac index.
